# Sex-dependent VEGF expression underlies variations in human pluripotent stem cell to endothelial progenitor differentiation

**DOI:** 10.1038/s41598-019-53054-z

**Published:** 2019-11-13

**Authors:** Lauren N. Randolph, Xiaoping Bao, Michael Oddo, Xiaojun Lance Lian

**Affiliations:** 1 Department of Biomedical Engineering, Pennsylvania State University, University Park, PA 16802 USA; 2 Huck Institutes of the Life Sciences, Pennsylvania State University, University Park, PA 16802 USA; 3 Department of Biology, Pennsylvania State University, University Park, PA 16802 USA; 4 Davidson School of Chemical Engineering, Purdue University, West Lafayette, IN 47907 USA

**Keywords:** Cell biology, Pluripotent stem cells

## Abstract

Human pluripotent stem cells (hPSCs) offer tremendous promise in tissue engineering and cell-based therapies because of their unique combination of two properties: pluripotency and a high proliferative capacity. To realize this potential, development of efficient hPSC differentiation protocols is required. In this work, sex-based differences are identified in a GSK3 inhibitor based endothelial progenitor differentiation protocol. While male hPSCs efficiently differentiate into CD34 ^+ ^CD31^+^ endothelial progenitors upon GSK3 inhibition, female hPSCs showed limited differentiation capacity using this protocol. Using VE-cadherin-GFP knockin reporter cells, female cells showed significantly increased differentiation efficiency when treated with VEGF during the second stage of endothelial progenitor differentiation. Interestingly, male cells showed no significant change in differentiation efficiency with VEGF treatment, but did show augmented early activation of VE-cadherin expression. A sex-based difference in endogenous expression of VEGF was identified that is likely the underlying cause of discrepancies in sex-dependent differentiation efficiency. These findings highlight the importance of sex differences in progenitor biology and the development of new stem cell differentiation protocols.

## Introduction

In the regenerative medicine field, topics such as personalized medicine, immunoengineering, and stem cell therapies are frequently discussed; however, cell sex variations are rarely considered despite prevalent evidence of sex differences in many different diseases, such as cardiovascular disease, autoimmune disease, Alzheimer disease, and diabetes^1–6^. Many of these conditions involve malfunction or attack of terminally differentiated senescent cells making them excellent candidates for the development of stem cell-based therapies. Efforts in the stem cell biology field to derive these somatic cell types have progressed substantially, and in some cases, have produced efficient differentiation strategies^7–9^.

Endothelial progenitors and vascular endothelial cells, arising from the mesodermal germ layer, are of great interest in a variety of regenerative medicine, tissue engineering, and other research applications. One of the predominant challenges in the field of tissue engineering is vascularization in solid tissues. This requires efficient generation of endothelial progenitor cells that yield cells of the vasculature and would provide relevant materials for *in vitro* models of diseases in which endothelial cells play an important role, such as cardiovascular disease^10–14^. Additionally, endothelial progenitors with definitive hematopoietic potential are of great importance in developing directed differentiation strategies towards hematopoietic stem cells and blood cell components for therapeutic applications, such as hematopoietic stem cell transplant and T-cell based cancer therapies^15–17^. Directed differentiation strategies applied to human pluripotent stem cells (hPSCs) provide an infinite cell source as hPSCs can be propagated indefinitely while still retaining the capacity to differentiate into all manner of somatic cell types^18,19^. Furthermore, genetic disease context can be introduced using induced pluripotent stem cells (iPSCs) derived from relevant patient populations^20,21^. hPSCs also provide a platform for developing model systems, using genome editing technology such as CRISPR-Cas9, to elucidate the cellular signals involved in differentiation to a somatic cell type^22–26^.

A variety of directed differentiation strategies for deriving endothelial cells from hPSCs have been established, including chemically defined small molecule based protocols^27–33^. This protocol efficiently produces endothelial progenitor cells from hPSCs via GSK3 inhibition to activate the Wnt signaling pathway. Using this strategy, it is possible to efficiently generate CD34^ + ^CD31^ + ^VE-cadherin^+^ endothelial progenitor cells from male hPSCs; however this differentiation strategy proved inefficient for the differentiation of female hPSCs^27,28^. While it is common practice to perform experiments on a variety of cell lines to validate new findings or protocols, it is less common to specifically compare differences that might exist as a function of a cell’s sex, and the sex of a cell line is often not reported. In fact, many protocols discussing directed differentiation to a somatic cell type of interest use all male or all female cell lines^10,15,27,34^. In order to generate clinically useful cells with universal applicability, a directed differentiation method that works efficiently for both male and female cells is crucial.

No studies have been done with hPSCs to show that a differentiation strategy might result in different outcomes or differentiation efficiencies with relation to cell sex. Some research has shown sex differences in differentiated somatic cell types. For example, variance in skeletal muscle regeneration capacity and the oxidative stress response of male and female muscle-derived stem cells have been identified^35^. Sex hormones and receptors have also been shown to affect only female hematopoietic stem cell self-renewal^36^. Distinctions in proliferation of smooth muscle progenitor cells derived from hPSCs have also been shown as a function of cell sex in addition to disparity in extracellular matrix protein expression in these cells; however, no difference in differentiation efficiency was observed^37^. Differences in autosomal gene expression between male and female hPSCs have been reported, suggesting that male and female cells could respond differently to the same differentiation stimuli^38^. The identification of sex-based variations in response to differentiation cues would provide critical insight into the function of sex in endothelial progenitor development. This could have broad implications in progenitor cell biology as well as the development and implementation of therapeutic products using the vascular and hematopoietic cells that are derived from this progenitor stage.

Many methods, currently used to generate endothelial progenitors for further differentiation to hemogenic or endothelial lineages, rely on the use of VEGF among other growth factors^10,37,39,40^. Based on previous experiments, the addition of Sunitinib, a VEGF receptor inhibitor, at any stage of the endothelial progenitor differentiation will result in abrogation of the endothelial progenitor population, which highlights the importance of endogenous VEGF pathway in endothelial progenitor differentiation^27^. Therefore, this paper studied the hypothesis that addition of VEGF to the GSK3 inhibitor-based protocol^27^ could enhance female hPSC differentiation to endothelial progenitors.

## Results

### Generation and validation of VE-cadherin knockin hPSC lines

**Figure 1:**
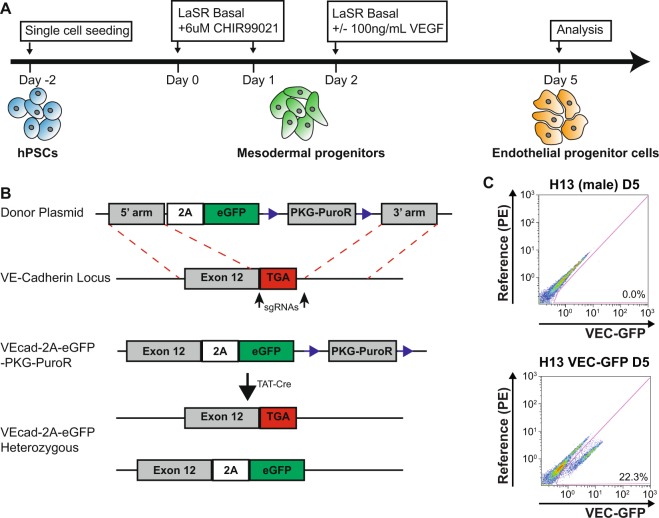
Generation and validation of male and female hESC VE-cadherin knockin reporter cell lines (**A**) Schematic of the endothelial progenitor differentiation from hPSCs. (**B**) Design for the VEC-GFP KI where a 2A-GFP sequence was inserted before the stop codon of VEC and a heterozygous KI single cell derived clone was isolated. (**C**) Flow cytometry analysis showing the separation of a GFP+ population in Day 5 endothelial progenitors derived from H13 VEC-GFP KI cells compared to endothelial progenitors derived from wild type H13 cells.

Several VE-cadherin (VEC)-GFP knockin (KI) reporter hPSC clones were generated to allow easy and antibody free isolation, quantification, and tracking of cells successfully differentiated to endothelial progenitors (Fig. 1A). A donor plasmid was designed with the desired insert, eGFP, preceded by a 2A sequence and followed by a floxed PGK-Puro^R^ cassette, and the 5′, and 3′ homology arms based on the sequence immediately before and after the VEC stop codon respectively (Fig. 1B). After electroporation, single cell derived colonies were isolated and tested to identify successful heterozygous KI clones.

To validate the function of the VEC-GFP KI cells, male H13 VEC-GFP KI cells and wild type H13 cells were differentiated to endothelial progenitor cells without VEGF in 5 days. Flow cytometry analysis was then used to evaluate the expression of GFP and obtained ~22% of the KI cells expressing VEC-GFP while no GFP positive population was observed with the wild type cells as expected (Fig. 1C). This efficiency is consistent with previous reports.

### VEGF supplementation enables generation of endothelial progenitor cells from female hPSCs

**Figure 2:**
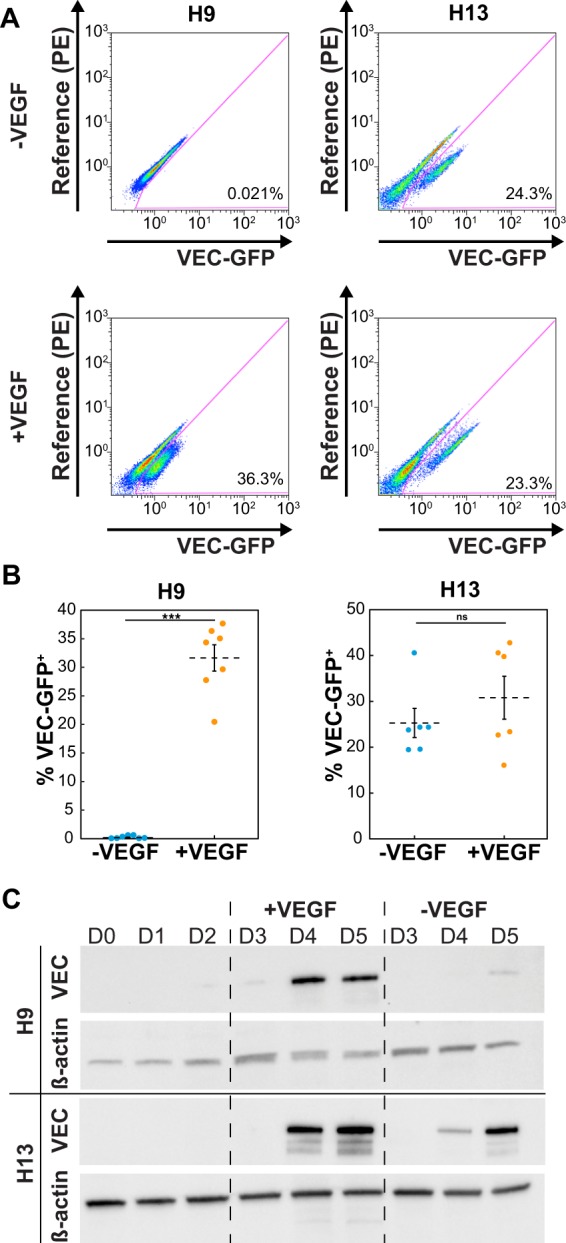
Effects of VEGF supplementation on endothelial progenitor differentiation in male and female cells (**A**) Representative flow cytometry analysis results showing VEC-GFP expression in H9 (female, left column) and H13 (male, right column) VEC-GFP KI cells after endothelial progenitor differentiation with (bottom row) or without (top row) VEGF (**B**) Quantification of flow cytometry analysis where each punctum represents an individual experiment (N = 7 for both conditions with H9 and N = 6 for both conditions with H13, ***p < 0.001). (**C**) Western blot showing the expression of VEC as the cells differentiate with or without VEGF for H9 and H13 cells. β-actin is a housekeeping control.

To determine whether supplementation with VEGF during endothelial progenitor differentiation would increase differentiation efficiency for female hPSCs, H9 (female) and H13 (male) VEC-GFP KI cells were differentiated with or without 100 ng/mL VEGF_165_ added into the media from day 2 to day 5. Whereas GFP positive cells were detected with or without VEGF_165_ treatment for male H13 hPSCs, GFP positive cells were only detectable with VEGF_165_ treatment for H9 cells (Fig. 2A). To quantify changes in efficiency of the differentiation, the percentage of the day 5 cells that were expressing GFP was analyzed using flow cytometry from a minimum of 6 independent differentiations. The addition of VEGF_165_ allowed the emergence of a GFP+ endothelial progenitor population in H9 cells totaling approximately 36% of the cells as compared to <0.1% without VEGF_165_ (Fig. 2A,B). The differentiation efficiency obtained by adding VEGF_165_ with the female cells is comparable to the originally and currently reported efficiencies obtained with male cells, if not higher^10,27^. Furthermore, statistical analysis via two-tailed student’s t-test showed there was a statistically significant difference in the percentage of the GFP+ population between the H9 cells differentiated with and without VEGF_165_ (p < 0.001). For wild type H9 cell differentiation with VEGF_165_ treatment, similar differentiation efficiency was achieved, which highlights that electroporation and KI procedures do not affect differentiation (Supplementary Fig. S1). For male H13 cells, there was no statistically significant difference between the two conditions (with or without VEGF_165_) (Fig. 2A,B). Statistical analysis with a two-way ANOVA and a Bonferroni post hoc test showed that both the sex of the cells and interaction between the cell sex and VEGF_165_ presence produced statistically significant effects on the differentiation efficiency (p < 0.001). All flow plots, including controls, are shown in Supplementary Fig. S2.

To evaluate the effects of VEGF_165_ supplementation on the temporal kinetics of VEC expression, a western blot was performed using cell lysate collected from cells during each day of endothelial progenitor differentiation from day 0–5. β-actin protein content was used as a loading control (Supplementary Fig. S3). Without VEGF_165_, male H13 hPSCs showed strong VEC expression on day 5 as well as weaker expression on day 4 whereas female cells showed barely detectable VEC expression on day 5 (Fig. 2C). However, upon the addition of VEGF_165_, strong VEC expression is detected on both day 4 and 5 in female H9 cells and a notable increase in day 4 VEC expression is seen in male H13 cells (Fig. 2C).

### Endogenous VEGF expression varies based on a cell’s sex

**Figure 3:**
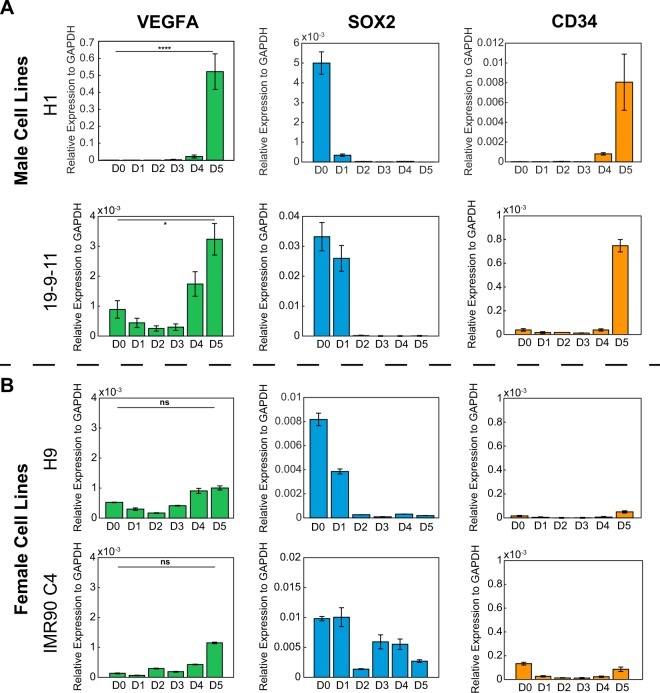
Analysis of endogenous VEGF expression in male and female cells during endothelial differentiation (**A**) qPCR results from two male cell lines: 6-9-9 (male iPSCs) and 19-9-11 (male iPSCs), and (**B**) two female cell lines: H9 (female hESCs) and IMR90C4 (female iPSCs), showing expression of VEGF, SOX2, and CD34 over the course of endothelial progenitor differentiation. Student’s T-test was performed to evaluate statistical significance between D0 and D5 expression for VEGF and is indicated on the plot. (ns: not significant, *p < 0.05, ****p < 0.0001).

Based on the effects of VEGF supplementation on the kinetics of VEC expression and previous assumptions that endogenously secreted VEGF_165_ in male hPSCs was sufficient to promote endothelial progenitor differentiation, endogenous VEGF_165_ expression levels were quantified for comparison. RNA was collected from each day of the differentiation performed without VEGF supplementation, from hESC lines H1 (male), H9 (female), and iPSC lines 19-9-11 (male), IMR90C4 (female). Bright field images of H1 and H9 cell differentiation were taken daily during endothelial progenitor differentiation (Supplementary Fig. S4). qPCR analysis was performed to quantify the relative expression of VEGF_165_, SOX2 (a pluripotency marker), and CD34 (an endothelial progenitor marker). Both male and female cells showed decreasing expression of SOX2 over the course of the differentiation from D0 to D5, which was expected due to loss of pluripotency (Fig. 3A). The male cells also showed a spike in CD34 expression on D5 (Fig. 3A), indicating successful derivation of endothelial progenitor cells and aligning with previous reports showing CD34 expression over the course of this differentiation^27^. Interestingly, the male cells showed an increase in endogenous VEGF expression over the course of the differentiation, peaking on day 5 with a statistically significant increase over the day 0 expression level (Fig. 3A). In contrast, the female cells showed diminished activation of VEGF expression and no statistically significant increase over the day 0 expression level for both female hESCs and iPSCs (Fig. 3B). Additional comparison of the expression level of VEGF_165_ on day 5 between male and female cells showed that the male cells have significantly higher expression than the female cells at this point in the differentiation (p < 0.0001).

## Discussion

In order to better understand the causes underlying sex-based differences in endothelial progenitor differentiation from hPSCs, VEC-GFP KI hPSCs were generated and used to show that supplementation with VEGF causes a significant increase in efficiency of endothelial progenitor differentiation in female cells increasing from less than 1% to 36%, but shows no significant change in differentiation efficiency with male cells. Furthermore, the addition of VEGF augmented the existing activation of VEC in male cells by increasing expression on Day 4 and replicated this trend in female cells. In contrast, female cells without VEGF showed no activation on day 4 and minimal VEC expression on day 5 indicative of differences in cellular signaling activation related to VEGF. However, no premature emergence of VEC expression is observed with VEGF_165_ supplementation indicated that the addition of VEGF_165_ is augmenting signaling pathways already activated as opposed to deviating from the desired developmental program.

To elucidate the cause of this difference, endogenous VEGF_165_ expression was quantified. The data revealed a statistically significant difference in endogenous VEGF expression on day 5 male and female cells. This represents the first report of a sex difference in response to *in vitro* stem cell differentiation stimulus.

Efficiencies obtained for this differentiation (<50%) do not indicate that the most effective means of deriving endothelial progenitors has been identified; there may be additional signaling pathways that require activation or inhibition to better promote the endothelial progenitor cell fate. However, identification of intrinsic differences between male and female hPSCs that receive the same treatment and stimulation are of importance for both the design of research studies and downstream therapeutic development. Many groups performing stem cell research use both male and female cells in their work. However, it is not always a standard that is met. Biased research using male or female cells can result in the development of therapeutics with inherent tailoring to one sex. Understanding differences between the sexes is also a key point of study in the developmental biology. Here, a select number of hPSC lines have been examined, and this may in turn limit the broader applicability of these results. Further in-depth study of this phenomenon in more hPSC lines, as it applies to both downstream differentiation from endothelial progenitors and other developmental lineages, and careful consideration of its effect on experimental design could lead to long sought solutions to elusive directed differentiation strategies.

In summary, the addition of VEGF to the previously established differentiation protocol overcomes identified efficiency differences in male and female hPSC differentiation to endothelial progenitor cells. While VEGF is often added in endothelial differentiation protocols, evidence is provided for a sex-based variation in its effect on differentiation efficiency. Taken together, the data point to intrinsic differences in VEGF signaling between male and female cells in response to the same endothelial progenitor differentiation method. This work augments the growing body of work highlighting the importance of evaluating the role of sex in stem cell differentiation, particularly when generating cells with immediate clinical application.

## Methods

### Maintenance of hPSCs

Human pluripotent stem cells (Female: H9 hESCs, IMR90C4 iPSCs; Male: H13 and H1 hESCs, 19-9-11 iPSCs) were maintained on Matrigel (Corning)-coated plates in LaSR pluripotent stem cell medium according to previously published methods^28,41,42^. LaSR pluripotent stem cell media does not contain any sex steroids. All cell culture experiments involving human pluripotent stem cell lines were approved by the Embryonic Stem Cell Oversight Committee at the Pennsylvania State University and carried out in accordance with the approved guidelines. All hPSC lines were obtained from WiCell. Informed consent was obtained from all subjects.

### Construction of *CDH5* donor plasmid and sgRNA

Donor plasmid construction and sgRNA cloning was performed as previously described^43^. Briefly, DNA fragments located before and after the stop codon of CDH5 approximately 2 kb in length were PCR amplified from genomic DNA and cloned into OCT4-2A-eGFP-PKG-Puro (This plasmid was a gift from Rudolf Jaenisch; Addgene #31938), replacing the OCT4 homology arms. pSpCas9(BB)-2A-Puro(PX459)V2.0 (This plasmid was a gift from Feng Zhang; Addgene #62988) was digested with BbsI and one of two sgRNAs targeted up- and downstream of the CDH5 stop codon (sgRNA1: TCAGCCAGCATCTTAAACCTGGG and sgRNA2: TTTTTGGAGGCTGTGGTGCCTGG) were inserted.

### Electroporation

H13 and H9 hPSCs were treated with 10 μM ROCK inhibitor (Y27632) for 3 to 4 hours prior to electroporation. Cells were digested by Accutase at 37 °C for 10 min and 2.5–3 million singularized cells were electroporated with 3 μg gRNA1, 3 μg gRNA2, and 6 μg CDH5‐2A‐eGFP donor plasmids in 200 μl cold PBS using the Gene Pulser Xcell System (Bio‐Rad) at 320V, 200 μF, and 1,000 Ω in a 0.4 cm cuvette. After electroporation, the cells were cultured in mTeSR1 and underwent drug selection using puromycin to remove any unmodified cells. The cells were then plated as single cells and allowed to form single cell-derived colonies. Colonies were picked and PCR genotyping was used to identify a successful heterozygous knock in clone. These cells were then treated with Cre recombinase to remove the puromycin resistance cassette, and single cell colony selection was performed again to isolate a heterozygous clone with successful excision of PGK-Puro^R^.

### Endothelial progenitor differentiation of hPSCs

Cells were differentiated to the endothelial progenitor state as previously described^27,28^. Briefly, 50,000 cells/cm^2^ were seeded onto a Matrigel-coated plate in LaSR pluripotent stem cell medium supplemented with 5 μM Y27632 (Selleckchem) on day -2. From day 0–1, the differentiation was initiated by changing media with LaSR basal medium supplemented with 6 μM CHIR99021 (Selleckchem). On day 2 the media was changed a final time with LaSR basal medium and some conditions were supplemented with 100 ng/mL VEGF_165_ (PeproTech). Cells were analyzed on day 5.

### Flow cytometry analysis

Cells were dissociated into single cells with Accutase for 10 min at 37 °C and then added at a 1:2 v/v ratio to DPBS with 0.5% BSA. Data were collected on a Beckman Coulter FC500 flow cytometer or a BD Accuri C6 cytometer and analyzed using FlowJo. Gating was done based on the corresponding untreated cell control.

### Western blotting

Cells were washed with DPBS and lysed with Mammalian Protein Extraction Reagent (Thermo Fisher) with 1X Halt’s Protease and Phosphatase (Thermo Fisher) by incubation for 3 minutes. Cell lysate was collected and stored at −80 °C until used. Samples were mixed with Laemmli sample buffer (BioRad) at a working concentration of 1X and incubated at 97 °C for 5 minutes. Samples were loaded into a pre-cast MP TGX stain free gel (BioRad) and run at 200V for 30 min in 1X Tris/Glycine/SDS buffer (BioRad). Protein was transferred to a PVDF membrane using a Trans-blot Turbo Transfer System (BioRad). The membrane was blocked for 30 minutes at room temperature in 1X TBST+ 5% Dry Milk. The membrane was incubated overnight at 4 °C with primary antibodies and for 1 hour at room temperature with secondary antibodies (Supplementary Table 1) in 1X TBST+ 5% Dry Milk. The membrane was washed between each antibody exposure with 1X TBST. Chemiluminescence was activated using Clarity Western ECL Substrate (BioRad) and the blot was imaged using a ChemiDoc Touch Imaging System and Image Lab software (BioRad).

### Quantitative PCR (qPCR)

RNA was extracted from the cells on each day of differentiation using a Direct-zol RNA MiniPrep Plus Kit (Zymo Research R2071). A Maxima First Strand cDNA Synthesis kit (Thermo Fisher K1641) was used to generate cDNA. An Applied Biosystems QuantStudio3 was used for performing qPCR with Power SYBR Green PCR Master Mix (Applied Biosystems 4367659) and primers (Supplementary Table 2). Each sample was run in triplicate. Data was analyzed by normalizing target gene C_t_ values to GAPDH C_t_ values. Relative expression to GAPDH was set to zero in the event that no measurable gene expression was detected.

### Statistics

Data obtained from multiple experiments or replicates are shown as the mean ± standard error of the mean. Where appropriate, Student’s *t* test or two-way ANOVA were utilized (alpha = 0.05) with a Bonferroni post hoc test. Data were considered significant when p < 0.05. Statistical tests were performed using custom MATLAB scripts.

## Supplementary information

Supplementary Info

## Data Availability

The data sets obtained and used in this study are available upon request submitted to the corresponding author. The scripts used for data analysis in this study are available upon request submitted to the corresponding author.
