# Supplementary material for: Sex-dependent VEGF expression underlies variations in human pluripotent stem cell to endothelial progenitor differentiation
Source: Sci Rep. 2019 Nov 13;9:16696. doi: 10.1038/s41598-019-53054-z (PMC6853961; doi:10.1038/s41598-019-53054-z)
Supplement: Supplementary file 1 — Supplementary Info [file 41598_2019_53054_MOESM1_ESM.pdf]

# Sex-dependent VEGF expression underlies variations in human pluripotent stem cell to endothelial progenitor differentiation

Lauren N. Randolph[1,2], Xiaoping Bao[4], Michael Oddo[1], Xiaojun Lance Lian[1,2,3,*]

[1]Department of Biomedical Engineering, Pennsylvania State University, University Park, PA, 16802, USA,

[2]Huck Institutes of the Life Sciences, Pennsylvania State University, University Park, PA, 16802, USA,

[3]Department of Biology, Pennsylvania State University, University Park, PA, 16802, USA,

[4]Davidson School of Chemical Engineering, Purdue University, West Lafayette, IN, 47907, USA

* Correspondence should be addressed to: Xiaojun Lance Lian (Lian@psu.edu)

**Supplementary Table 1:** Antibodies used in immunostaining analysis.

| Antibody | Source | Application |
|---|---|---|
| CD144-FITC | Miltenyi Biotec, recombinant human IgG1, Clone: REA199, Cat#: 130-100-742 | 1:25 (FC) |
| VE-cadherin | Santa Cruz, mouse igG1, Clone: F-8, Cat#: sc-9989 | 1:1000 (WB) |
| β-actin-HRP | Cell Signaling Technology, rabbit, Clone: 13E5, Cat#: 5125S | 1:20000 (WB) |
| Secondary Antibody | Alexa 488 Goat anti Mouse IgG1, Cat#: A-21121 | 1:1000 (IF) |
| Secondary Antibody | Anti-mouse IgG HRP-linked, Cell Signaling Technology, Cat#: 7076S | 1:1000 (WB) |

**Supplementary Table 2:** Primers used in qPCR experiments.

| Primer | Sequence | Size (bp) / $T_m$ (°C) / No. of Cycles |
|---|---|---|
| GAPDH_FWD | 5’-GTGGACCTGACCTGCCGTCT-3’ | 152 / 55 / 40 |
| GAPDH_REV | 5’-GGAGGAGTGGGTGTCGCTGT-3’ | |
| VEGF_FWD | 5’-GGCAAAGTGAGTGACCTGCT-3’ | 106 / 55 / 40 |
| VEGF_REV | 5’-CTGTCTGTCTGTCCGTCAGC-3’ | |
| SOX2_FWD | 5’-CAAGATGCACAACTCGGAGA-3’ | 300 / 55 / 40 |
| SOX2_REV | 5’-GTTCATGTGCGCGTAACTGT-3’ | |
| CD34_FWD | 5’-CCTAAGTGACATCAAGGCAGAA-3’ | 201 / 55 / 40 |
| CD34_REV | 5’-GCAAGGAGCAGGGAGCATA-3’ | |

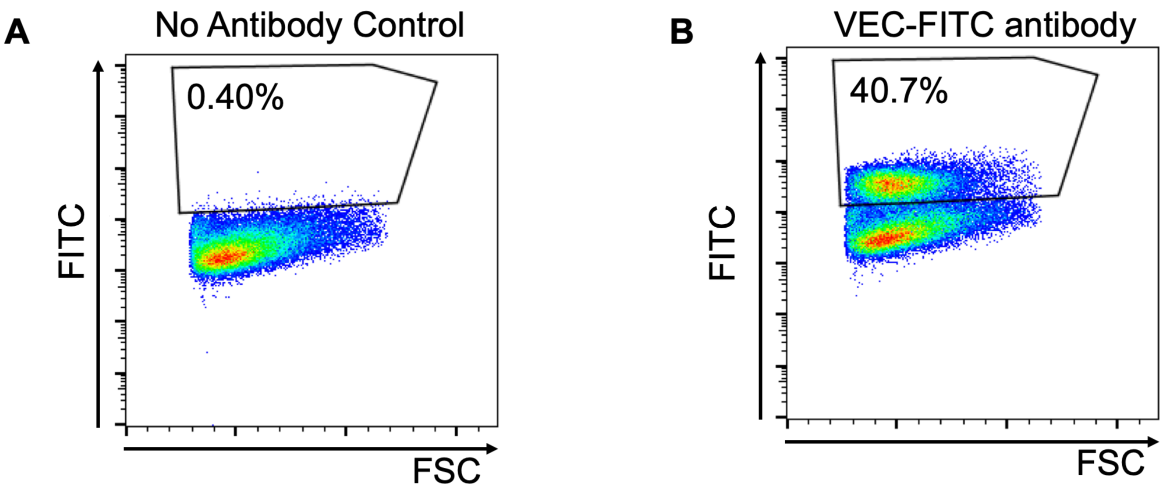


**<u>Figure S1</u>: Flow cytometry analysis for D5 endothelial progenitor cells derived from WT H9 cells.** H9 cells were differentiated using a GSK3 inhibitor based endothelial progenitor differentiation protocol (Gi protocol). On day 5 of differentiation, cells were analyzed for VEC expression by flow cytometry. (A) No antibody control (B) VEC-FITC antibody.

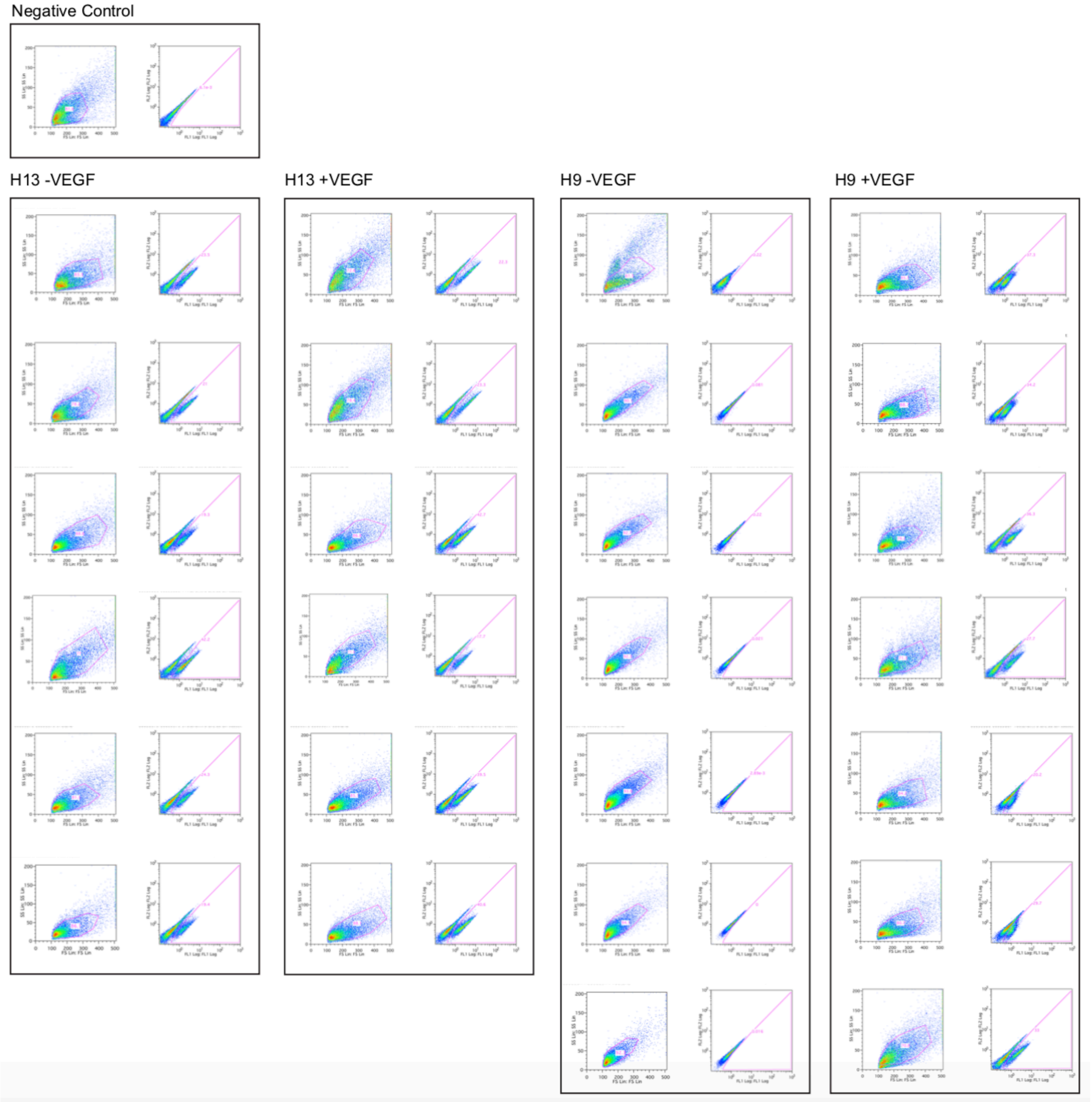


**<u>Figure S2:</u> Gating for all flow plots included in data set.** H9 and H13 VEC-GFP KI cell lines were differentiated using Gi protocol. On day 5 of differentiation, GFP expression was analyzed via flow cytometry.

A B

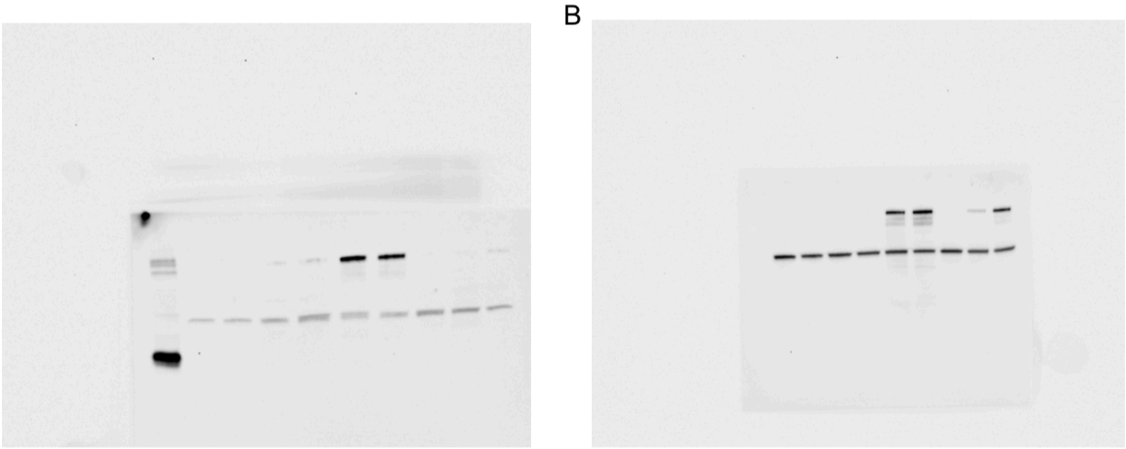

**<u>Figure S3:</u> Full blots for Western blot data.** A) H9 cells B) H13 cells were differentiated with Gi protocol. VEC and beta-actin expression was analyzed via western blot.

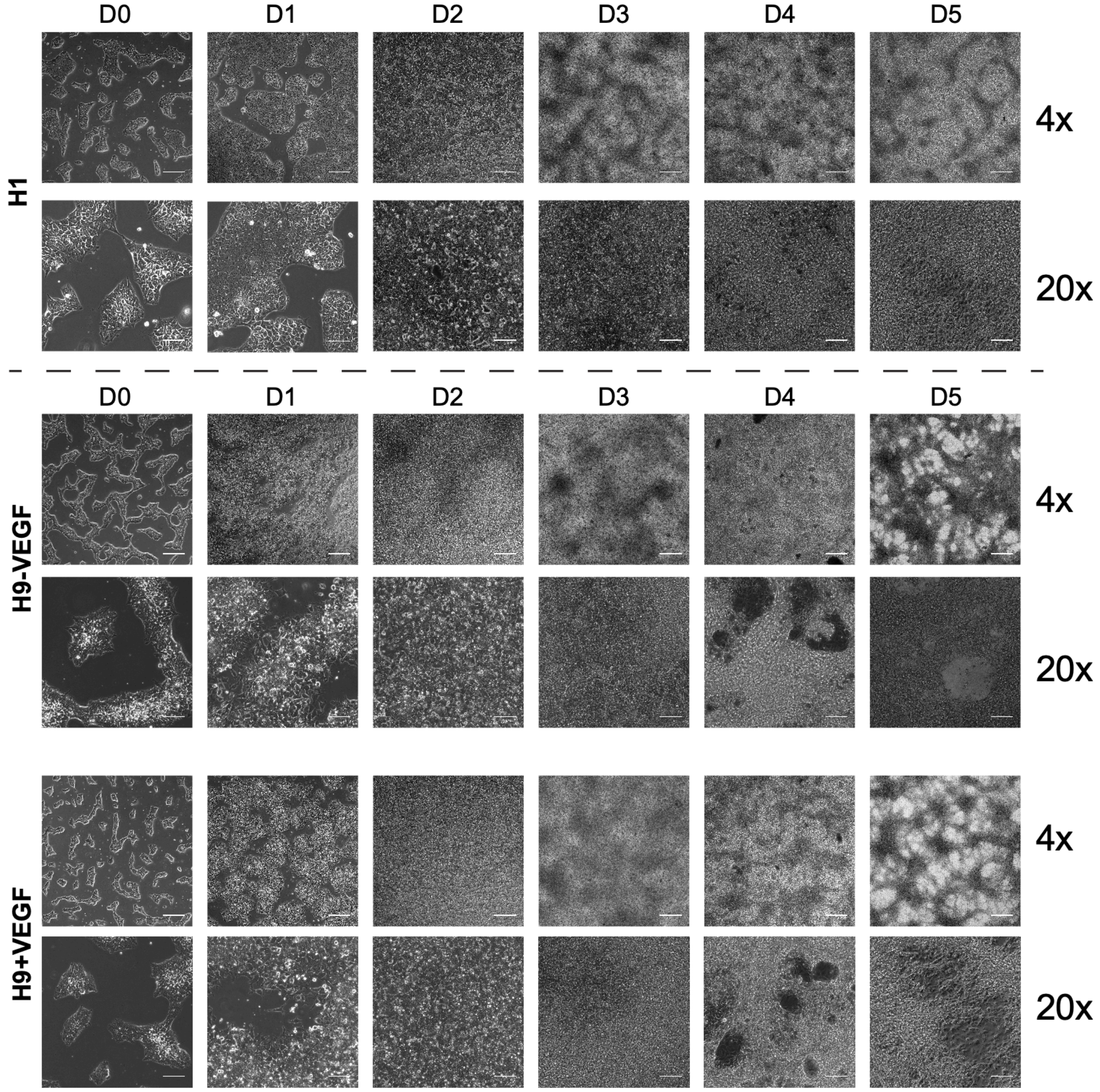


**<u>Figure S4:</u> Representative bright field images of differentiation.** H1 and H9 cells were differentiated with Gi protocol. Bright field images were taken daily during differentiation. Scale bars for 4x images are 500 μm. Scale bars for 20x images are 100 μM.
